# Relationship of Visceral Adipose Tissue With Dilated Perivascular Spaces

**DOI:** 10.3389/fnins.2020.583557

**Published:** 2021-02-04

**Authors:** Yunli Qi, Mengqi Lin, Yunjun Yang, Yanxuan Li

**Affiliations:** ^1^Department of Radiology, The First Affiliated Hospital of Wenzhou Medical University, Wenzhou, China; ^2^Department of Radiology, Wenzhou Hospital of Traditional Chinese Medicine Affiliated to Zhejiang Chinese Medical University, Wenzhou, China; ^3^School of Pharmaceutical Sciences, Wenzhou Medical University, Wenzhou, China

**Keywords:** cerebral small vessel disease, abdominal obesity, visceral adipose tissue, MRI, dilated perivascular space

## Abstract

**Background:**

Dilated perivascular spaces (dPVS) are considered to be a type of cerebral small vessel disease (CSVD) as well as an important part of the glymphatic system. Although obesity has been shown to play a significant role in the development of CSVD, there are no studies addressing the correlation between obesity and dPVS. We aimed to study the relationship between abdominal fat distribution and dPVS in neurologically healthy cohorts.

**Methods:**

A total of 989 subjects, who were examined during a health examination project, were included in this study. We measured both visceral adipose tissue (VAT) and subcutaneous adipose tissue (SAT) areas using abdominal computed tomography. The dPVS scores were also evaluated in the basal ganglia (BG) and the centrum semiovale (CSO).

**Results:**

In a multivariate ordinal regression analysis, the relationship between VAT area and CSO-dPVS scores remained significant (β [95% confidence interval {CI} = 0.00003395] [0.00001074–0.00005716], *P* = 0.004), especially in male cohorts (β [95% CI] = 0.00004325 [0.00001772–0.00006878], *P* = 0.001) after adjusting for age; sex; and glucose, creatinine, uric acid, high-density lipoprotein, and low-density lipoprotein levels, while no association was found between SAT area and dPVS scores. The effects of quartile VAT area on CSO-dPVS were also significant in male cohorts (odds ratio [95% CI] = 1.33 [1.139 – 1.557], *P* < 0.001).

**Conclusion:**

We demonstrated a positive association between VAT and CSO-dPVS scores in a healthy cohort, which was more prominent in males.

## Introduction

The spaces surrounding small blood vessels in the brain are termed perivascular spaces. These include the periarteriolar, pericapillary, and perivenular spaces (Wardlaw et al., 2020). Perivascular spaces are the most important part of the brain glymphatic drainage system and function as a communication network between cellular fluid and cerebrospinal fluid to remove soluble proteins and other metabolic-waste-containing macromolecules such as glucose, lipids, and amino acids (Tarasoff-Conway et al., 2015; Bacyinski et al., 2017). It has been reported that reduced function of the glymphatic system leads to accumulation of toxic proteins or metabolic waste in the brain and results in a series of inflammatory reactions, leading to the occurrence of diseases such as Parkinson’s and Alzheimer’s diseases (Zou et al., 2019). When compensatory or pathologic enlargement occurs in perivascular spaces of the brain due to an impairment in the clearance of the glymphatic system in such a microenvironment, it manifests as dilated perivascular space (dPVS) and can be detected by both T1- and T2-weighted magnetic resonance imaging (MRI). To date, dPVS is considered a type of cerebral small vessel disease (CSVD), and the pathology of dPVS remains unclear.

Obesity is defined as the accumulation of excess fat and has been shown to play a significant role in the development of cerebrovascular diseases (Keys, 1980; Feinleib, 1985; Kannel, 1985; Wajchenberg, 2000). So far, the effect of obesity on the development of CSVD has been well established. Like white matter hyperintensity, lacunae and microbleeding are also associated with obesity as detected by imaging measurements (Karcher et al., 2013; Yamashiro et al., 2014). In addition, animal studies provide some insight on the potential impact of obesity on the occurrence of CSVD. For example, a previous study (Virdis et al., 2019) suggested that the pathology of endothelial dysfunction caused by obesity might be related to oxidative stress, inflammation, or enzyme-mediated pathways and may later manifest as CSVD. Interestingly, visceral adipose tissue (VAT) and subcutaneous adipose tissue (SAT) have different effects on disease formation (Gil et al., 2011). For example, a study demonstrated that VAT had a significant effect on atherosclerosis in obese patients, while SAT did not (Fantuzzi and Mazzone, 2007). Although numerous studies have been conducted on CSVD and obesity, the relationship between dPVS and obesity is not clear.

In this study, we focused on the relationship between abdominal fat distribution that was measured directly by abdominal computed tomography (CT), and dPVS in healthy cohorts. We also conducted laboratory examinations to predict factors that might lead to the formation of dPVS. We hypothesized that accumulation of adipose tissue might result in the formation of dPVS in one or the other pathological way.

## Materials and Methods

### Subjects

**FIGURE 1:**
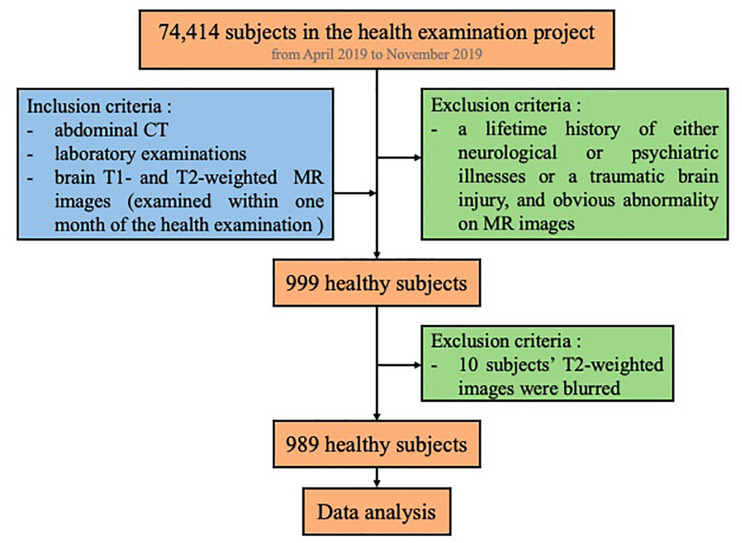
Subject screening workflow.

We screened all the subjects (74,414) who participated in the health examination project at The First Affiliated Hospital of Wenzhou Medical University from April 2019 to November 2019. Among these subjects, we first searched for subjects whose examinations included abdominal CT images in the PACS system as well as laboratory examinations on the same day (*N* = 25,364). Furthermore, we searched for those who had brain MR examination within the following month. Also, we excluded subjects with a lifetime history of either neurological or psychiatric illnesses or a traumatic brain injury, as well as persons with obvious abnormality on their brain MRI (such as malformations and space-occupying lesions, infarcts, hematomas, and heterotopic white matter), leading to a total of 999 persons. Additionally, as MR images of 10 subjects were blurred, they were not included in the study. Finally, we included 989 subjects in this study (Figure 1). Our study was approved by the Medical Ethics Committee of The First Affiliated Hospital of Wenzhou Medical University. Since this was a retrospective cross-sectional study including both males and females, written informed consent for each subject was waived by the Medical Ethics Committee.

### Assessment of Factors Predictive of dPVS Formation

All 989 subjects underwent T1- and T2-weighted MRI, abdominal CT, and laboratory examinations for detecting the levels of the following: total protein (TP), urea nitrogen (UN), glucose, creatinine, triglycerides, total cholesterol (TC), high-density lipoprotein (HDL), low-density lipoprotein (LDL), and homocysteine.

The dPVS was defined and assessed using both axial T1- and T2-weighted images according to the Standards for Reporting Vascular Changes on Neuroimaging criteria by two trained neuroradiologists (YQ and YL with 7 and 4 years of experience, respectively) who were blinded to the clinical information. The dPVS had high intensity as seen in T2-weighted images, low intensity in T1-weighted images, and low intensity in fluid-attenuated inversion recovery images. It was necessary to distinguish the lacunar infarcts from dPVS, which are generally oval-shaped lesions greater than 3 mm. According to Doubal et al. (2010), a 4-point visual rating scale (0 = no dPVS, 1 = less than 10 dPVS, 2 = 11–20 dPVS, 3 = 21–40 dPVS, and 4 = greater than 40 dPVS) was used to evaluate dPVS in the centrum semiovale (CSO) area and the basal ganglia (BG). After examining all relevant levels, the score was assigned as per the abovementioned visual rating scale. The two brain hemispheres were evaluated separately, and the higher score was used as the subject’s final dPVS score. The dPVS scores of all the 989 participants were evaluated by the two radiologists. The inter-rater reliability was excellent for BG-dPVS scores (intraclass correlation coefficient = 0.80) and CSO-dPVS scores (intraclass correlation coefficient = 0.85). The dPVS rating scale of a senior radiologist was used for analysis. We obtained broad MR image acquisitions as follows: 1) T1-weighted images (repetition time [TR]/echo time [TE] = 2005/15 ms) and 2) T2-weighted images (TR/TE = 4900/120 ms).

We calculated SAT and VAT using CT images of the abdomen at the horizontal level of the third lumbar vertebra. Based on the specifications by Kim et al. (2017), we identified the tissues within the parietal peritoneum, except the spine and paravertebral muscles, as VAT, and the tissues outside the fascia of the abdominal wall muscles as SAT.

**FIGURE 2:**
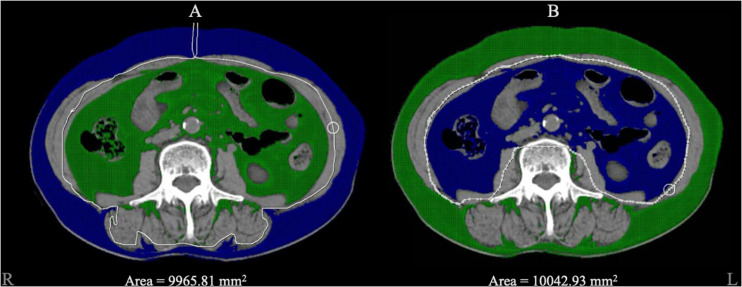
The diagram of adipose tissue segment. Blue area represented the selected zone within the CT value range from –150 to –40 Hounsfield units. **(A)** The blue area represented for the subcutaneous adipose tissue we circled, and 9965.81 mm^2^ was automatically calculated as the area. **(B)** The blue area represented for visceral adipose tissue and 10042.93 mm^2^ was automatically calculated as the area.

We used post-processing software (version 4.6; GE Healthcare) to set the CT value range from –150 to –40 Hounsfield units, automatically marked the tissue within the CT value range on the image level of the third lumbar vertebra and manually circled the SAT and VAT areas to finally show the output of SAT and VAT areas. Figure 2 shows CT images of the third lumbar vertebral level of a subject and the SAT and VAT area ranges. CT scans were performed using a standard clinical protocol: tube voltage = 120 kV(p), automatically adjusted tube current, and axial section thickness = 5 mm.

### Statistical Analysis

First, the data are shown as the mean ± standard deviation of the normally distributed continuous variables (determined by the Kolmogorov–Smirnov test) and the number of binary variables. In addition, the values were presented separately for males and females.

Second, the associations between dPVS scores and possible predictors were analyzed by univariate ordinal regression analysis.

Third, multivariate ordinal regression analysis with dPVS scores as the dependent variable was performed to assess the association with VAT area and VAT/SAT ratio separately, by adjusting for variables with *P* < 0.05 in univariate linear regression.

Certain previous studies demonstrated that the adipocyte activity appeared to be rather different between males and females (Fox et al., 2004; Porter et al., 2009; Bouchi et al., 2015). To confirm this sexual difference regarding the effects of adipose tissues on dPVS scores, we performed stratified multivariate ordinal regression analysis by sex. The adjusted variables were determined using univariate ordinal regression analysis. On the basis of the results of sex-stratified multivariate ordinal regression analysis, we divided the VAT area into 4 grades according to quartiles (Degree 1: VAT area < 9621.5 mm^2^; Degree 2: 9621.5 mm^2^ < VAT area < 14056.5 mm^2^; Degree 3: 14056 mm^2^ < VAT area < 18920.5 mm^2^; Degree 4: VAT area > 18920.5 mm^2^) to show the correlation clearly.

SPSS version 25 (IBM SPSS, Chicago, IL, United States) was used for all statistical analyses in this study. *P* < 0.05 was considered statistically significant.

## Results

The demographic data of 989 subjects are shown in Table 1. The mean BG-dPVS and CSO-dPVS scores (± SD) were 1.01 ± 0.12 and 1.42 ± 0.666, and the mean areas of SAT and VAT (± SD) were 12177 ± 4914 mm^2^ and 12043 ± 6910.2 mm^2^, respectively.

**TABLE 1:** Demographic characteristics of the cohort.

Index	Total		Female		Male	
	Mean	SD	Mean	SD	Mean	SD
Number, n	989		339		650	
Age	45.2800	7.8860	45.8700	8.3560	44.9700	7.6100
TP (g/L)	78.2520	51.7000	79.4800	60.0100	77.5950	46.6840
UN (mmol/L)	5.0310	1.7363	4.7690	2.4507	5.1710	1.1676
Glucose (mmol/L)	5.1520	1.5193	5.0810	1.8079	5.1900	1.3395
Creatinine (μmol/L)	68.3500	13.3490	55.7100	7.1040	75.1200	10.7290
UA (μmol/L)	352.5300	130.3800	279.8600	61.9580	391.4400	140.4100
Triglycerides (mmol/L)	1.8778	1.6622	1.3197	0.9658	2.1466	1.8681
TC (mmol/L)	5.3374	1.0477	5.2411	1.0160	5.3890	1.0615
HDL (mmol/L)	1.2292	0.3344	1.4063	0.3762	1.1344	0.2651
LDL (mmol/L)	2.9884	0.8051	2.8926	0.7746	3.0397	0.8169
Homocysteine (μmol/L)	11.3500	4.3720	9.6000	2.2690	12.2900	4.9070
BG-dPVS scores	1.0100	0.1200	0.9900	0.1210	1.0100	0.1180
CSO-dPVS scores	1.4200	0.6660	1.3100	0.5620	1.4800	0.7090
SAT area (mm^2^)	12177.0000	4914.0000	14158.0000	5105.0000	11117.0000	4464.6000
VAT area (mm^2^)	12043.0000	6910.2000	7490.1000	4297.7000	14480.0000	6813.0000
VAT/SAT	1.0792	0.7704	0.5312	0.2642	1.3723	0.7924

*TP, total protein; UN, urea nitrogen; UA, uric acid; TC, total cholesterol; HDL, high-density lipoprotein; LDL, low-density lipoprotein; BG, basal ganglia; CSO, centrum semiovale; dPVS, dilated perivascular spaces; VAT, visceral adipose tissue; SAT, subcutaneous adipose tissue.*

In univariate regression analysis (Table 2), CSO-dPVS scores were significantly associated with age; sex; levels of glucose, creatinine, UA, HDL, and LDL; VAT area; and VAT/SAT ratio. Meanwhile, BG-dPVS scores were significantly associated with age, sex, creatinine levels, VAT area, and VAT/SAT ratio. Tolerance (TC) = 0.095 and variance inflation factor (VIF) = 10.577 were collinear with other indicators; hence, they were not considered in this study.

**TABLE 2:** Univariate ordinal regression analysis between dPVS scores and demographic, laboratory and radiological factors.

Parameters	Collinearity diagnostics		CSO-dPVS scores		BG-dPVS scores	
	T	VIF	β (95% CI)	*P*	β (95% CI)	*P*
Age	0.928	1.078	0.038 (0.021 to 0.054)	< 0.001*	0.002 (0.001 to 0.003)	0.001*
Sex	0.365	2.741	−0.487 (−0.772 to 0.203)	0.001*	0.023 (0.006 to 0.039)	0.008*
TP (g/L)	0.992	1.008	0.001 (−0.002 to 0.003)	0.624	−0.000004347 (0 to 0)	0.956
UN (mmol/L)	0.942	1.061	0.022 (−0.049 to 0.093)	0.548	0.001 (−0.004 to 0.006)	0.671
Glucose (mmol/L)	0.903	1.107	0.102 (0.011 to 0.181)	0.011*	0 (−0.005 to 0.005)	0.94
Creatinine (μmol/L)	0.470	2.126	0.012 (0.002 to 0.022)	0.014*	0.001 (0 to 0.001)	0.032*
UA (μmol/L)	0.763	1.311	0.001 (0 to 0.002)	0.017*	0.00003737 (0 to 0)	0.229
Triglycerides (mmol/L)	0.760	1.317	0.111 (−0.011 to 0.234)	0.075	0 (−0.005 to 0.004)	0.858
HDL (mmol/L)	0.722	1.385	−0.686 (−0.122 to 0.251)	0.002*	−0.006 (−0.03 to 0.018)	0.641
LDL (mmol/L)	0.937	1.067	0.177 (0.017 to 0.338)	0.031*	0.002 (−0.008 to 0.012)	0.721
Homocysteine (μmol/L)	0.888	1.126	−0.011 (−0.041 to 0.020)	0.505	0 (−0.002 to 0.002)	0.877
SAT area (mm^2^)	0.274	3.652	0.00001736 (−0.000007566 to 0.00004229)	0.172	0.00000004395 (0 to 0)	0.956
VAT area (mm^2^)	0.461	2.170	0.00005266 (0.00003389 to 0.00007144)	< 0.001*	0.000001245 (0 to 0)	0.034*
VAT/SAT	0.365	2.737	0.301 (0.140 to 0.462)	< 0.001*	0.011 (0.001 to 0.022)	0.03*

*P < 0.05. The *P*-value level chosen to determine significance was established to be 0.05. TP, total protein; UN, urea nitrogen; UA, uric acid; HDL, high-density lipoprotein; LDL, low-density lipoprotein; BG, basal ganglia; CSO, centrum semiovale; dPVS, dilated perivascular spaces; VAT, visceral adipose tissue; SAT, subcutaneous adipose tissue.

After adjusting for age; sex; and levels of glucose, creatinine, UA, HDL, and LDL, the association between CSO-dPVS scores and VAT area remained significant (*P* = 0.004), while no association was found between BG-dPVS scores and adipose tissue indicators after adjusting for age, sex, and creatinine levels (Table 3).

**TABLE 3:** Associations between dPVS scores and adipose tissue area.

Index	All				Male				Female			
	VAT area (mm^2^)		VAT/SAT		VAT area (mm^2^)		VAT/SAT		VAT area (mm^2^)		VAT/SAT	
	β (95% CI)	*P*	β (95% CI)	*P*	β (95% CI)	*P*	β (95% CI)	*P*	β (95% CI)	*P*	β (95% CI)	*P*
CSO-dPVS scores^a^	0.00003395 (0.00001074–0.00005716)	0.004*	0.058 (–0.136 to 0.252)	0.558	0.00004325 (0.00001772 to 0.00006878)	0.001*	0.101 (–0.093 to 0.295)	0.306	–0.00001485 (–0.00008473 to 0.00005504)	0.067	–0.977 (–2.088 to 0.135)	0.085
BG-dPVS scores^b^	0.00002297 (–0.00005732 to 0.000106)	0.575	0.206 (–0.371 to 0.783)	0.484	0.00002159 (–0.00006301 to 0.000106)	0.617	0.21 (–0.343 to 0.763)	0.457	0.00001151 (–0.000239 to 0.000262)	0.928	–0.211 (–4.128 to 3.706)	0.916

*P < 0.05. The *P-*value level chosen to determine significance was established to be 0.05. ^*a*^In this statistical model, age, sex, glucose, creatinine, UA, HDL, and LDL were set as covariates. ^*b*^In this statistical model, age, sex, and creatinine were set as covariates. dPVS, dilated perivascular spaces; VAT, visceral adipose tissue; SAT, subcutaneous adipose tissue; BG, basal ganglia; CSO, centrum semiovale.

In the sex-stratified multivariate ordinal regression analysis, only significant association was found only between VAT area and CSO-dPVS scores of males (*P* = 0.001). When the VAT area data were divided into 4 parts, the OR (95% CI) value was 1.33 (1.139–1.557) (*P* < 0.001) between VAT area levels and CSO-dPVS scores of males.

## Discussion

In this study, we demonstrated that the VAT area was positively associated with CSO-dPVS scores in a neurologically healthy cohort, while SAT did not show a positive association. Because other obesity parameters showed no statistical significance, VAT was acknowledged as the most potent predictor of dPVS scores in our cohort. Furthermore, this association was more prominent in male subjects.

The exact underlying pathophysiologic mechanisms of the relationship between adipose tissue and dPVS are not clear. The positive association of VAT area and CSO-dPVS scores can be attributed to the following reasons. First, obesity is thought to be a systemic disease that affects every cell in the body through endocrine, metabolic, and inflammatory activities of adipocytes (Mazurek et al., 2003; Raggi, 2013). Large amounts of VAT in the body have been linked to cerebrovascular risk factors in numerous studies (Finelli et al., 2013). Second, the basic pathology of CSVD has been reported to originate from vascular endothelial cells (Wardlaw et al., 2019), while vascular endothelial cells are also affected by pro-inflammatory cytokines secreted by adipocytes, such as plasminogen activator inhibitor type-1, tumor necrosis factor-alpha, and interleukin 1 and 6 (Schafer and Konstantinides, 2011). In addition, some studies suggested that VAT mainly secreted pro-inflammatory factors, while SAT mainly secreted beneficial factors, such as leptin (Ibrahim, 2010). This explains why we found a negative correlation between SAT and dPVS scores in the ordinal regression analysis, although it was not statistically significant. The finding of a positive association between CSO-dPVS and VAT area has not been reported in previous studies, but it has been reported that other types of CSVD such as white matter hyperintensity, lacunae, and microbleeding (Karcher et al., 2013; Yamashiro et al., 2014) are related to VAT, so we believe that our findings are consistent with the conclusions of previous literature.

Interestingly, the effects of VAT were more prominent in males than in females. Sex differences were always a confounding factor. The findings of the present study might be explained by the difference of fat distribution in males and females (Golan et al., 2012; Bouchi et al., 2015). Also, males tend to accumulate fat at the visceral depot at any age (Matsuzawa et al., 1995) which causes males to have more VAT than females. Moreover, as previously discussed, VAT, as an endocrine organ (Wajchenberg, 2000), had more negative effects than SAT, which may lead to vascular endothelial damage (Virdis et al., 2019).

Despite the novel findings of this study, there were some limitations. First, there were differences in the proportion of males and females, and although we corrected for the sex factor in the statistical model, the non-linear effect might still be present as a confounding factor. Second, this study was a cross-sectional analysis, so we were unable to provide evidence of causality. Further prospective studies are needed to identify the underlying pathophysiological mechanisms. Finally, our study was designed as a single-center, retrospective observational study. Analyses were limited to persons with no obvious abnormalities on their brain MRI (participants with brain infarcts or hematomas were excluded). Hence, our study population is not representative of the general population in this age range, and a selection bias cannot be excluded.

## Conclusion

We demonstrated a positive association between VAT and CSO-dPVS scores in a healthy cohort, which was more prominent in males. Considering that VAT had a significant association with various risk factors associated with the development of dPVS, we speculated that obese men might have poorer clearance of the glymphatic system in the brain, and further animal experiments are needed to confirm it.

## Data Availability Statement

The raw data supporting the conclusions of this article will be made available by the authors, without undue reservation.

## Ethics Statement

The studies involving human participants were reviewed and approved by the Medical Ethics Committee of The First Affiliated Hospital of Wenzhou Medical University. Written informed consent from the patients/participants are not required to participate in this study in accordance with the national legislation and the institutional requirements.

## Author Contributions

YL and YY directed the experiment’s overall design and revised the manuscript. YQ and ML collected the data. YL and YQ evaluated the image data. YL conceived of the design’ details and analytic plan, then performed the statistical analyses, and drafted the manuscript. All authors contributed to the article and approved the submitted version.

## Conflict of Interest

The authors declare that the research was conducted in the absence of any commercial or financial relationships that could be construed as a potential conflict of interest.

## Abbreviations

CSVD: cerebral small vessel disease
CT: computed tomography
dPVS: dilated perivascular spaces
HDL: high-density lipoprotein
LDL: low-density lipoprotein
MR: magnetic resonance
SAT: subcutaneous adipose tissue
TC: total cholesterol
TP: total protein
UN: urea nitrogen
VAT: visceral adipose tissue.
